# Immunity to Laser Power Variation in a DFB Diode Laser Based Optical Gas Sensor Using a Division Process

**DOI:** 10.3390/s150409582

**Published:** 2015-04-22

**Authors:** Hengtai Chang, Jun Chang, Qingjie Huang, Qiang Wang, Changbin Tian, Wei Wei, Yuanyuan Liu

**Affiliations:** School of Information Science and Engineering and Shandong Provincial Key Laboratory of Laser Technology and Application, Shandong University, Jinan 250100, China; E-Mails: Hunter_chang@126.com (H.C.); qjhuang@sdu.edu.cn (Q.H.); iamwq1989@gmail.com (Q.W.); changbin_tian@163.com (C.T.); zero931103056@126.com (W.W.); lyy12956@163.com (Y.L.)

**Keywords:** optical gas sensor, division process, residual amplitude modulation, wavelength modulation spectroscopy, unstable laser power

## Abstract

The division process used in a DFB diode laser-based optical gas sensor was studied to improve the immunity to laser power variation. Residual amplitude modulation (RAM) in wavelength modulation spectroscopy (WMS) detection was eliminated by intensity normalization using a division process. As a result the detected harmonic signals showed a significant improvement in line shape. For the first harmonic (1*f*) signal, Bias was improved from 38.7% to 1.2%; Baseline Difference was improved from 2.7% to 0.69% and Asymmetry was improved from 15.4% to 0.22%. For the second harmonic (2*f*) signal, the Asymmetry Coefficient was improved from 103% to 5.1%. Moreover the division process can further suppress the influence of unstable laser power. As a result, for the 1*f* signal, stable detection with a variation coefficient of 0.59% was obtained over a wide dynamic range (0.38–8.1 mW). For the 2*f* signal, stable detection with a variation coefficient of 0.53% was obtained from 0.64 mW to 8.27 mW. The test results showed a good agreement with the theoretical analysis and the proposed method has considerable potential application in gas sensing.

## 1. Introduction

Trace gas qualification analysis is needed in a wide variety of applications, ranging from methane monitoring in coal mines [1], water vapor monitoring in high voltage transmission equipment [2], to ammonia detection in environmental science [3]. Because of the advantages of safety in hazardous environments, remote detection capability and immunity to electromagnetic radiation, spectroscopic measurement of gas concentrations has emerged as a potential method for gas concentration measurement. The molecules of many chemical species of interest (ammonia, carbon dioxide, carbon monoxide, methane, water vapor *etc*.) exhibit strong absorbances in the mid-infrared region of the spectrum [4]. However the need for cooled lasers causes difficulties with deployment in some applications [5]. The near-infrared spectra are typically overtones of the fundamental absorption lines in the mid-infrared region and can be significantly weaker, but the availability of high quality light sources, primarily derived from telecommunication applications, can counteract this disadvantage. Because of high spectral-power density and narrow line width, near-infrared distributed feedback (DFB) diode lasers have seen widespread utilization in optical gas sensing design [1,2,5,6]. When using spectroscopic measurements for gas concentration, the transmitted light attenuates as the incident light propagates through the gas. However, the very information concerning the gas concentration is the laser light energy absorbed by the probed gas molecules. 

For DFB diode laser-based optical gas sensors, although direct detection [2,7,8] is simple and easy to implement, wavelength modulation spectroscopy (WMS) [9,10,11,12] is commonly used for efficient noise reduction and high sensitive detection. For direct detection the absorption signal is small, and sits on a high background (*i.e.*, the absorbed laser light relative to the transmitted light). Especially when the gas concentration is at the ppmv level, the absorption is too small to determine. Usually a normalization technique is used to extract the small absorption signal from the high background noise [13]. For WMS detection, the small absorption signal can be figured out more easily. Because there is no harmonic signal of the absorption line in the high background noise, the high background signal can be eliminated to obtain the small absorption signal [14].

Because of the modulation characteristics of DFB diode laser, the laser intensity changes as the driving current changes. In addition to the wavelength modulation of the DFB diode laser, residual amplitude modulation (RAM) appears at the receiver, thus the harmonic signal can inevitably be affected. Besides the effect on the RAM, the output laser power is potentially unstable during on-site applications because of temperature and pressure variations. Note that the laser power variation in this paper comprises the RAM during laser modulation and laser power instability deriving from ambient environmental changes. In the following, a division process is applied to DFB diode laser-based optical gas sensing. The performance of RAM elimination in WMS detection and the effects of unstable laser power suppression are studied. The effect of the division process is demonstrated by measuring the 1368.597 nm absorption line of a H_2_O/air mixture.

## 2. RAM Suppression in WMS Detection

### 2.1. Harmonic Signals Analysis

For gas detection, direct detection can easily recover the absolute absorption line shape of the probed gas molecules. However the scan frequency of direction detection is usually tens of Hertz. Compared with direction detection, WMS detection enables detection at frequencies of a few thousands of Hertz. The choice of a higher frequency can apparently suppress laser and 1/*f* noise, and the use of a lock-in amplifier can further reduce the noise outside the bandwidth [5]. Hence WMS detection is more sensitive than direction detection by one to two orders of magnitude. Usually the driving current comprises a slow ramp current and a fast sinusoidal current [10]. The ramp slowly scans the center frequency *ν*_1_ (cm^−1^) through the absorption line at a typical rate of a few Hertz to tens of Hertz. The fast sinusoidal driving current translates to a wavelength modulation.

Because of the laser power *versus* current characteristics of DFB diode laser, the above current applied to the diode laser produces a modulation in laser power along with the increasing ramp current and its sinusoidal modulation. Taking the modulation effect (including intensity and wavelength modulation) into account, the received light signal at the photoelectric detector can be expressed as follows [5]:

(1) $$I_{\text{signal}}=\left[I\left(\nu_{1}\right)+\Delta I\cos\left(\omega t+\varphi \right)\right]\left[1-\alpha \left(\nu_{1}+\delta \nu \cos\left(\omega t\right)\right)CL\right]$$

where *ν*_1_ (cm^−1^) is the center frequency of the laser light and *I*(*ν*_1_) is the corresponding intensity. Both are slowly changed by the ramp current. Δ*I* is the amplitude of RAM. *ω* (rad/s) is the modulation circular frequency by the sinusoidal driving current. *φ* is the phase shift between the power and the wavelength modulation. *α*(*v*) (cm^−1^) is the frequency (*ν*, cm^−1^) related absorption coefficient, *C* is the mole fraction of the probed gas in the mixed gas, *L* (cm) is the path length through which the laser beam and gas molecules interact. *δν* (cm^−1^) is the wavelength modulation amplitude.

The modulated absorption coefficient can be expressed in Fourier cosine series around ν_1_ [14]:

(2) $$\alpha \left(\nu \right)=\sum_{k=0}^{k=\infty }H_{k}\left(\nu_{1},\delta \nu \right)\cos\left(k\omega t\right)$$

The function of *H*_k_(*ν*_1_,*δν*) can be given by [14]:

(3) $$\begin{array}{l} H_{0}\left(\nu_{1},\delta \nu \right)=\frac{1}{2\pi }\int_{-\pi }^{+\pi }\alpha \left(\nu_{1}+\delta \nu \cos\mu \right)d\mu \\ H_{k}\left(\nu_{1},\delta \nu \right)=\frac{1}{\pi }\int_{-\pi }^{+\pi }\alpha \left(\nu_{1}+\delta \nu \cos\mu \right)\cos k\mu d\mu ,k>0 \end{array}$$

For convenience, *ωt* is substituted by *u* in Equation (3). Usually 1*f* and 2*f* detections are used to recovery the absorption signal. In practice, the RAM can inevitably distort the detected harmonic signals using WMS detection. In this case, the full expression for the detected 1*f* signal becomes:

(4) $$\begin{array}{ccc} I_{\omega } & = & \underset{1}{\underset{︸}{\Delta I\cos\left(\omega t+\varphi \right)}}-[\underset{2}{\underset{︸}{\Delta IH_{0}\left(\nu_{1},\delta \nu \right)\cos\left(\omega t+\varphi \right)}}\\ & & +\underset{3}{\underset{︸}{I\left(\nu_{1}\right)H_{1}\left(\nu_{1},\delta \nu \right)\cos\left(\omega t\right)}}+\underset{4}{\underset{︸}{\frac{1}{2}\Delta IH_{2}\left(\nu_{1},\delta \nu \right)\cos\left(\omega t-\varphi \right)}}]\times CL \end{array}$$

When obtained by a lock-in amplifier the first item, which can be referred to as concentration-independent RAM, leads to a high background. The second item and the fourth item are also from the RAM, and the detected 1*f* signal can be apparently distorted by the neighboring derivatives of the absorption coefficient. The third item could have been the ideal 1*f* signal. However, the intensity of *I*(*ν*_1_) varies with the ramp current or the scanning *ν*_1_. Thus the third item can also be distorted. Similarly the 2*f* signal has the following form:

(5) $$\begin{array}{ccc} I_{2\omega } & = & -[\underset{1}{\underset{︸}{\frac{1}{2}\Delta IH_{1}\left(\nu_{1},\delta \nu \right)\cos\left(2\omega t+\varphi \right)}}+\underset{2}{\underset{︸}{I\left(\nu_{1}\right)H_{2}\left(\nu_{1},\delta \nu \right)\cos\left(2\omega t\right)}}\\ & & \underset{3}{\underset{︸}{+\frac{1}{2}\Delta IH_{3}\left(\nu_{1},\delta \nu \right)\cos\left(2\omega t-\varphi \right)]}}\times CL \end{array}$$

Note again that, the first item and the third item are from the RAM. The detected 2*f* signal can be interrupted by the first and third derivatives. The second item, which could have been the ideal 2*f* signal, is also distorted by the *I*(*ν*_1_) variation. 

In brief the factors accounting for distorted harmonic signals are related to RAM and *I*(*ν*_1_) variation, which are shown as the first half of Equation (1). The differential technique division process can achieve common-mode rejection through normalizing the same interference factors of two signals. The reference light, which is shown as follows, is similar to the signal light in Equation (1) except for the absorption information:

(6) $$I_{\text{reference}}=I\left(\nu_{1}\right)+\Delta I\cos\left(\omega t+\varphi \right)$$

After the division process the inference factors accounting for distorted harmonic signals can be removed leaving:

(7) $$\begin{array}{l} \eta =\frac{I_{\text{signal}}}{I_{\text{reference}}}=\kappa \left[1-\alpha \left(\nu_{1}+\delta \nu \cos\left(\omega t\right)\right)CL\right] \end{array}$$

where *κ* is the ratio of *I*_signal_/*I*_reference_ away from any absorption. Likewise, based on Fourier analysis the 1*f* and 2*f* signals after the division process can be written as follows:

(8) $$\eta_{\omega }=\kappa H_{1}\left(\nu_{1},\delta \nu \right)\cos\left(\omega t\right)\times CL$$

(9) $$\eta_{2\omega }=\kappa H_{2}\left(\nu_{1},\delta \nu \right)\cos\left(2\omega t\right)\times CL$$

Comparing Equations (8) and (9) with Equations (4) and (5), it can be concluded that the absolute harmonic signals can be recovered without distortion after the division process.

### 2.2. Improvement Test with the Division Process

It can be seen from the above analysis that all the interference factors are from the change of laser intensity with the varying drive current. The most effective method to eliminate the interrupting effect is to stabilize the laser intensity. However, so far the technique of stabilizing the laser intensity is too expensive and not easy to achieve in practical application [3]. Here the interrupting effect can be eliminated by normalizing the laser intensity at the circuit level, which is realized through a divider.

**Figure 1:**
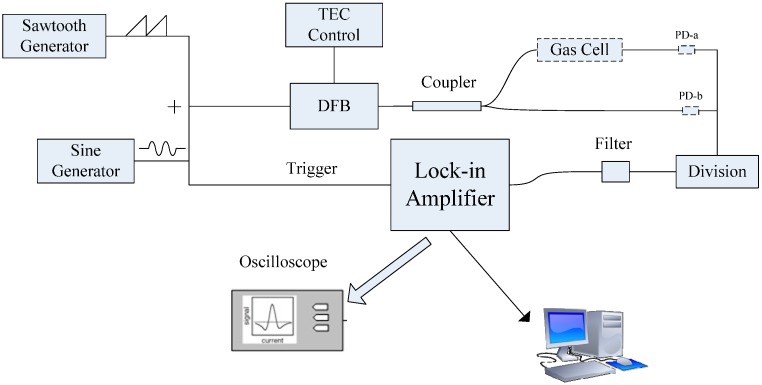
Schematic of WMS detection using a divider.

The test is based on a distributed feedback (DFB) diode laser for the detection of water vapor using the 1368.597 nm absorption line. As shown in Figure 1, a sawtooth current with a repetition rate of 30 Hz is added by a sinusoidal current with a frequency of 2 kHz. In addition a temperature controlling chip (LTC1923, Linear Technology, San Francisco, CA, USA) is used to control the Thermoelectric Cooler (TEC) of the DFB diode laser. The laser beam is split into two parts by a fused biconical taper coupler with a 1:1 splitting ratio. One beam directly couples on a photoelectric detector (named PD-b) and it is used as reference beam. The other beam couples on another photoelectric detector (named PD-a) after interacting with water vapor in the gas cell and it is used as the signal beam. The difference in optical path length for the two beams can be controlled within 5 cm and the effective length of the gas cell is 13 cm. The photocurrents of the two photoelectric detectors are transformed into voltage by two identical pre-amplifiers. Then the two voltage signals are processed by a division circuit. After a passive high-pass filter with a cutoff frequency of 5 Hz, the normalized voltage signal is introduced in a digital signal process lock-in amplifier (Model 7230, AMETEK, San Diego, CA, USA) to extract harmonic signals which are monitored using a oscilloscope in real-time. In addition, the voltage signal deriving from PD-a’s pre-amplifier is also introduced in 7230 to extract the harmonic signals, which are used as single-beam WMS detection to compare with the division process. Then the data is captured by a computer using a LabVIEW program.

In the following both the 1*f* and 2*f* signals are detected to verify the laser intensity normalization performance. During the test the detection phase of the lock-in amplifier is set as 0. Sample gas with a water vapor concentration of about 1200 ppmv is introduced into the gas cell. The 1*f* signals are obtained from the normalized voltage and the PD-a pre-amplifier successively.

**Figure 2:**
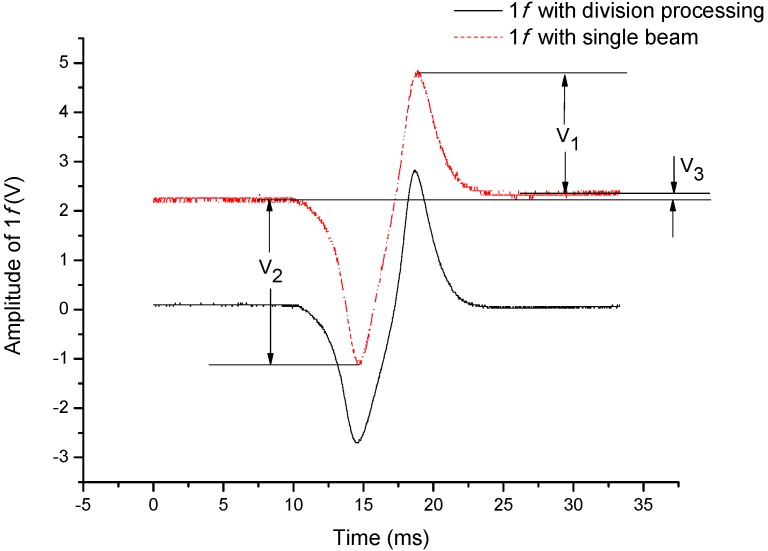
Comparison of 1*f* signals detected by the division process and single beam.

As is shown in Figure 2, the solid line “1*f* with division process” is from the normalized voltage and the dotted line “1*f* with single beam” is from the PD-a pre-amplifier.

It is apparent that the detected single-beam 1*f* signal has been seriously deformed. Three features are used to describe the deformed 1*f* signal. Firstly, an apparent bias voltage is added and we call it Bias here. Secondly, baselines on both sides are separated by a voltage difference (*V*_3_) in the profile and we call it Baseline Difference here. Thirdly, serious asymmetry exists in the profile, which appears in the difference of *V*_1_ and *V*_2_, and we call it Asymmetry here. In contrast the division process has significantly improved the detected 1*f* signal, which is labeled as “1*f* with division process” in Figure 2. The amplitude of the 1*f* signal is defined as the difference between the maximum and minimum. To reasonably show the improvement performance, the listed three features of the two detected 1*f* signals are normalized by their own amplitude. The comparison is listed in Table 1.

**Table 1:** Comparison of normalized 1*f*-profile features between the single-beam and division processes.

Normalized Features	Single Beam	Division Process
Bias	38.70%	1.20%
Baseline Difference	2.70%	0.69%
Asymmetry	15.40%	0.22%

**Figure 3:**
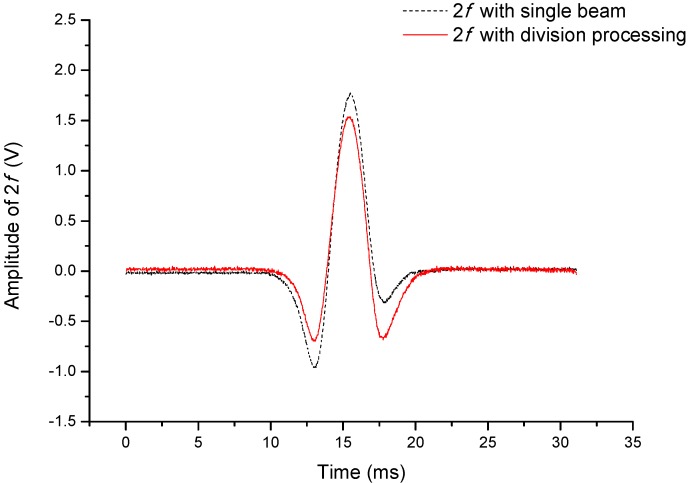
Comparison of 2*f* signals detected by the division and single beam processes.

The 2*f* signals are also obtained from the normalized voltage and the PD-a pre-amplifier successively. As is shown in Figure 3, the solid line “2*f* with division process” is from the normalized voltage and the dotted line “2*f* with single beam” is from the PD-a pre-amplifier. The most apparent feature of the deformed 2*f* profile is the asymmetry. It is shown in Figure 3 that the asymmetry of the detected 2*f* signal after the division process has been corrected significantly. To better show the improvement performance for the detected harmonic signals, an “Asymmetry Coefficient” is introduced. The asymmetry coefficient is defined as the difference of the two minima of the profile divided by the mean value of the two minima.

As a result, the symmetry has been improved from 103% for “2*f* with single-beam” to 5.1 % for “2*f* with division process”.

## 3. Laser Power Instability Suppression in WMS Detection

For the single-beam WMS detection shown by Equations (4) and (5), not only the driving current-induced RAM and variable *I*(*ν*_1_) can distort the detected harmonic signals, but also ambient environment-induced laser power instability can influence the amplitude of detected harmonic signals. In the following a test on immunity to laser power instability is carried out. The experiment platform mentioned in Figure 1 is further used in this test. The difference is that a variable optical attenuator (VOA) is inserted between the DFB diode laser and coupler. It is used to change the laser power substituting ambient environment-induced laser power instability.

**Figure 4:**
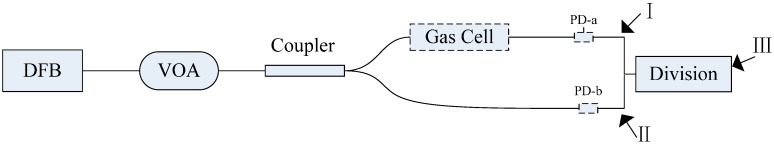
Simple schematic of test on immunity to laser power instability. Voltage at Ⅰ is used to obtain single-beam harmonic signal, averaged voltage at II is used to reflect laser power after VOA, voltage at III is used to obtain division-process harmonic signals.

To show the immunity to laser power instability, test results between single-beam harmonic signals and division-process harmonic signals are compared. In Figure 4 voltage at I is used to obtain single-beam harmonic signals and voltage at III is used to obtain division-process harmonic signals. Averaged voltage at II is obtained to indirectly reflect the laser power after VOA. As the splitting ratio of the coupler is 1:1, thus laser power after VOA can be figured out based on the averaged voltage at II and photoelectric conversion coefficient of PD-b.

**Figure 5:**
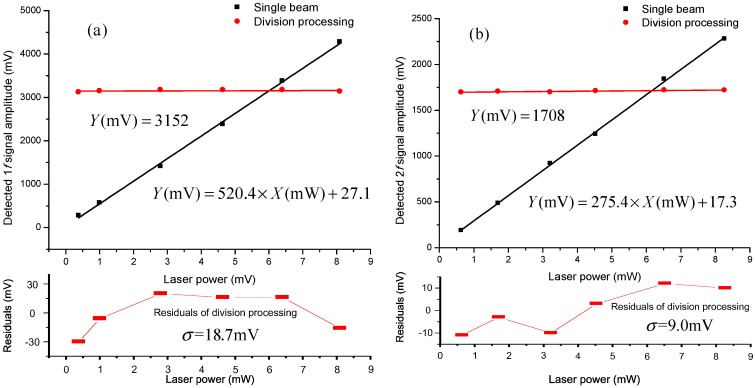
Test result of immunity to laser power instability. (**a**) is for the 1*f* signal and (**b**) is for the 2*f* signal.

Figure 5a shows the test result of immunity to laser power instability for the 1*f* signal. Laser power after VOA ranges from 0.38 mW to 8.1 mW. During the laser power adjustment the amplitude of the single-beam 1*f* signal is proportional to laser power. However the amplitude of the division-process 1*f* signal remains stable with a standard deviation of 18.7 mV and a corresponding variation coefficient of 0.59%. Figure 5b shows the test result for the 2*f* signal. During the adjustment, the laser power ranges from 0.64 mW to 8.27 mW. The amplitude of the single-beam 2*f* signal is also proportional to laser power and the amplitude of the division-process 2*f* signal remains stable with a standard deviation of 9.0 mV and a corresponding variation coefficient of 0.53%. Note that the variation coefficient is defined as the ratio of standard deviation to mean value.

The test results shown in Figure 5 indicate that the division process in WMS detection can significantly improve the immunity to laser power instability compared to single-beam WMS detection. This is of great importance during field applications, because laser power can be changed by many factors including temperature variation and fiber vibration.

## 4. Conclusions

In summary, a division process was studied in DFB diode laser-based gas sensors using WMS detection to improve the immunity to laser power variation (including RAM deriving from wavelength modulation and laser power instability deriving from ambient environment changes). The harmonic signals deformed by residual amplitude modulation were corrected by the division process. For the 1*f* signal, the Bias was reduced from 38.7% to 1.2%, the Baseline Difference was reduced from 2.7% to 0.69% and the Asymmetry was reduced from 15.4% to 0.22%. For the 2*f* signal, the Asymmetry Coefficient was reduced from 103% to 5.1%. Immunity to laser power instability has also been improved using the division process. As a result the amplitude of the 1*f* signal remained stable with a variation coefficient of 0.59% under the condition of an unstable laser power ranging from 0.38 mW to 8.1 mW. The amplitude of the 2*f* signal remained stable with a variation coefficient of 0.53% under the condition of an unstable laser power ranging from 0.64 mW to 8.27 mW.
